# Neutron imaging and molecular simulation of systems from methane and *p*-xylene

**DOI:** 10.1038/s41598-024-85093-6

**Published:** 2025-01-08

**Authors:** Martin Melčák, Tereza-Markéta Durďáková, Štěpán Tvrdý, Jonatan Šercl, Jong Min Lee, Pierre Boillat, Jan Heyda, Pavel Trtik, Ondřej Vopička

**Affiliations:** 1 Department of Physical Chemistry, University of Chemistry and Technology, Prague, Technická 5, 166 28 Prague 6, Czech Republic; 2 Laboratory for Neutron Scattering and Imaging, Paul Scherrer Institut, 5232 Villigen PSI, Switzerland; 3 Electrochemistry Laboratory, Paul Scherrer Institut, 5232 Villigen PSI, Switzerland

**Keywords:** Thermodynamics, Surface chemistry, Chemical physics, Thermodynamics, Imaging techniques

## Abstract

Bulk properties of two-phase systems comprising methane and liquid *p*-xylene were derived experimentally using neutron imaging and theoretically predicted using molecular dynamics (MD). The measured and predicted methane diffusivity in the liquid, Henry’s law constant, apparent molar volume, and surface tension compared well within the experimentally studied conditions (273.15 to 303.15 K, ≤ 100 bar). Since MD is a physical model, extrapolations of the two-phase systems properties were performed for a broader temperature range (260 to 400 K, ≤ 100 bar). Moreover, the species diffusivities in single phases formed by infinitely diluted *p-*xylene in methane were predicted under conditions relevant to the methane liquefaction (90 to 290 K, 50 bar). The predicted *p*-xylene diffusivity in the supercritical methane was one order of magnitude higher than that calculated using Wilke–Chang and He–Yu correlations. This study provides novel experimental and MD-simulated characteristics for this industrially relevant system, for which intensive freeze-out formation from the supercritical methane is predicted.

## Introduction

Benzene, toluene, ethylbenzene, xylenes (BTEX) and water are impurities of natural gas relevant to the formation of solids deposits (freeze-out) which can block devices in the processing and transportation^1–5^. Water and *p*-xylene are the most severe volatile contaminants due to the high temperature of normal melting and hydrate formation. Solid deposits are formed, for instance, at 172 bar and 276 K (methane hydrate^6^), or at 163 bar and 278 K (solid *p*-xylene)^4^. In this work, the focus is on the *p*-xylene – methane system. In recent studies, equilibrium conditions for the solid *p*-xylene formation have been determined experimentally and modelled using equations of states^1,4^. The intensity of the *p*-xylene freeze-out formation on the cold spots is presumably controlled by its diffusivity in the fluid. Understanding of not only the equilibrium condition but also freeze-out formation intensity can clearly contribute to the engineering of the natural gas purification and liquefaction devices^5^. 

As we have previously demonstrated, multiple system characteristics can be derived based on the recent *one-pot neutron imaging method*^7,8^of observing pressurized gas absorption into liquids, namely methane diffusivity, solubility, apparent volume, and interfacial energy. Molecular dynamics (MD) simulation is a physical model that enables the prediction of related system characteristics, such as diffusivity^9–11^, surface excess and interfacial energy^12^, partial molar volume^13^, and viscosity^11,14,15^. Clearly, the two independent methods (one experimental and one simulation-based), each of which necessitates single-component data only in this work, can be critically compared and complement each other. Besides that, MD can be used to study systems at wider ranges of conditions, and to provide experimentally hardly accessible quantities, such as partial molar volume or diffusivity of the major component.

In this work, we provide new experimental data for the two-phase system of *p*-xylene with methane using neutron imaging with focus on the region of supercooled liquid^4^. Based on the high difference of neutron cross-section between protium and deuterium^16^, neutron imaging enabled us to derive methane diffusivity in the liquid, apparent molar volume in the liquid, apparent Henry’s law constant, and surface tension from each experiment – multiple parameters are determined in *one pot*. This method represents an alternative to known chiefly single-purpose methods, such as the pendant drop method^17^, capillarity measurements^18^, methods based on sensing capillary waves^19,20^, and methods for the measurement of solubility, diffusivity, and density^21–24^. Thanks to low opacity of several engineering materials to neutrons, the neutron imaging is suited for investigations of pressurized systems. As a complement, the MD simulation model can be extrapolated to temperatures below and above the equilibrium condition of the solid *p*-xylene formation^4^, and was used for the prediction of *p*-xylene and methane diffusivity in supercritical and liquid methane at 50 bar and infinite *p*-xylene dilution. Thus, molecular-level models are used to predict the properties of highly supercooled liquids and fluids at industrially relevant conditions. Common predictive models for the diffusivity in diluted liquids^25,26^and supercritical fluids^26,27^ are used for comparison.

## Methods

### One pot neutron imaging

The neutron imaging experiments were conducted using a previously reported setup^7,8^ at the NEUTRA beamline^28^ at Paul Scherrer Institut at the measuring position No. 2 (L/D = 365). The setup contained a pair of equivalent axially symmetric titanium measuring cells placed in a duralumin block maintained at a constant temperature to within ± 0.1 °C using a Julabo F12-MA water circulator and sensed to within ± 0.1 K using a thermometer (Pt100, Greissinger GMH 3710), pressure was sensed using a transducer (Omega PXM409-100BAV). The cells were rinsed with acetone, vacuumed (< 0.01 Pa, Leybold D4B), and twice washed with fresh sample liquid prior to the filling. The cells were filled with the same liquid (*p*-C_8_D_10_, Table 1) thus providing two repeats for the measurement at each conditions. MIDI-box detector system using a 30 µm-thick Gd_2_O_2_S:Tb scintillator screen (RC-Tritec AG, Teufen, Switzerland) and a sCMOS camera (Andor Neo) fitted with a 100-mm objective (Zeiss Makro-Planar) were used, images of 2560 (W) × 2160 (H) pixels in size were collected with an isotropic pixel pitch of 21.59 µm, the spatial resolution is therefore estimated to be better than 80 µm. The acquisition scheme of the neutron radiographies consisted of several (usually seven) series of 50 images each of the 10 s acquisition time for each investigated system. For the evaluation of the data from the first two respective series, 10 data points were provided as an average of 10 images having the respective time stamp of the average time of the respective 10 images; for the latter series, the entire 50 images were averaged into a single data point having the time stamp of the average of the 50 images.

**Table 1:** Used gases and chemicals, initial purity as in the certificate of analysis by the supplier unless indicated otherwise.

Chemical	Supplier, initial purity
Methane (CH_4_)	Linde, 5.5, CAS 74–82-8
Nitrogen (N_2_)	PanGas, 5.0, CAS 7727–37-9 (purge gas – outer apparatus box)
*p*-xylene (*p*-C_8_D_10_)	Armar, 99.59 atom-% D, > 99.9 wt.%^#^, CAS 41051–88-1
Acetone	Penta, > 99.9 wt.% (rinsing agent)

^#^Chemical purity was not declared by the supplier and was determined using a GC–MS (Clarus 500, Perkin Elmer) with a capillary column containing Elite WAX ETR stationary phase (Perkin Elmer), value represents purity with respect to other C_8_ aromatic hydrocarbons.

**Fig. 1:**
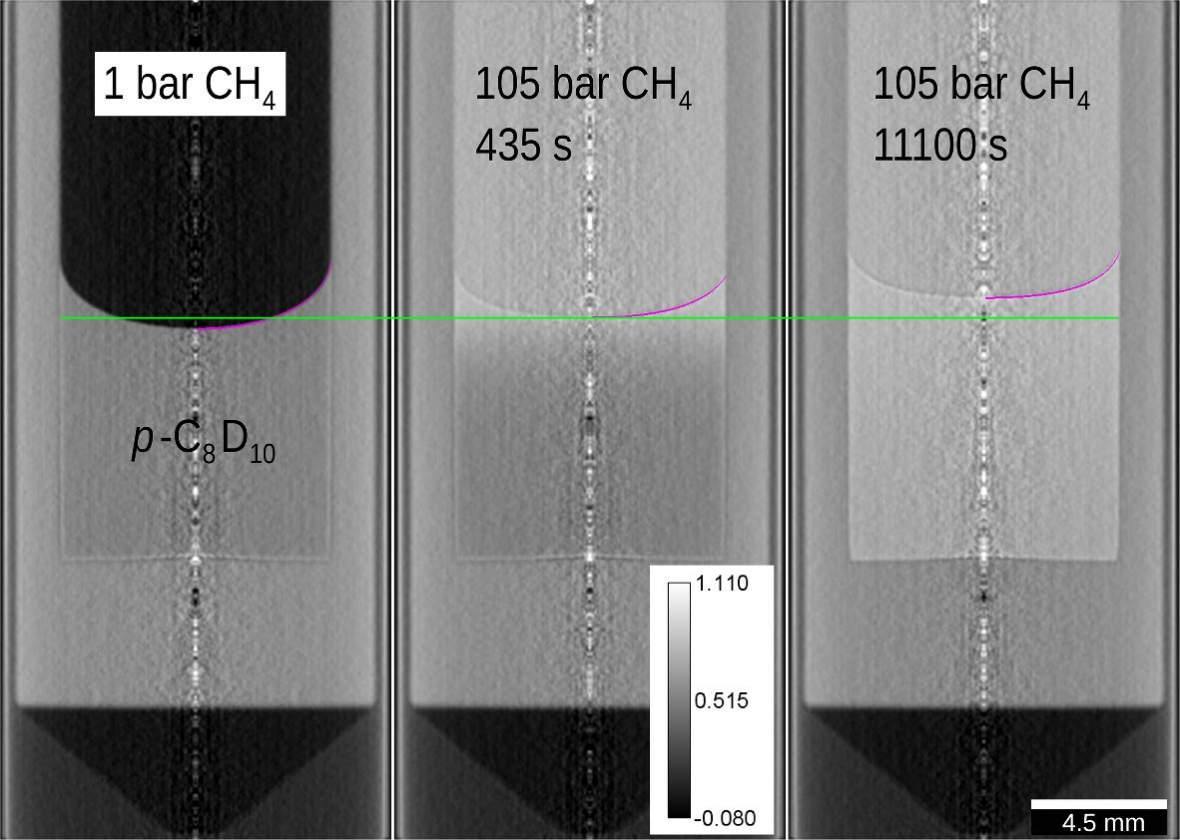
Central-plane tomographic reconstruction for cell with supercooled liquid *p*-C_8_D_10_ at 0.0 °C, methane pressure was increased at zero time. Gray value corresponds to the linear attenuation coefficient, inner diameter of the cell was 9.0 mm. Regression with solution of Eq. (10) is shown as purple curve, green curve is a reference line for the interface position.

Neutron radiographies of two perpendicular axially-symmetric test tubes (inner radius* R* = 4.5 mm) were acquired, each containing *p*-xylene equilibrated with methane at 1 bar. These tubes were subject to the methane pressure step, the diffusion of methane into the liquid was imaged. After applying filters and corrections^29,30^, the radiographs were reconstructed at the central plane of the sample via the onion-peeling algorithm^31^. The resulting tomographic reconstructions at the central plane of the sample (Fig. 1) are matrices of the overall linear attenuation coefficient (*Σ*) for the individual pixels.

### Theory

The overall attenuation by the binary mixture is contributed by the constituents, A (CH_4_) and B (*p*-C_8_D_10_). The contribution of B is negligible for the gaseous (supercritical) phase at the studied conditions^21,24^. For the liquid, concentration can thus be derived using the Beer-Lambert law

1 $${\text{ln}}\frac{{I^{0} }}{I} = \sigma_{{\text{A}}} N_{0} c_{{\text{A}}} d + \sigma_{{\text{B}}} N_{0} c_{{\text{B}}} d = \Sigma_{{\text{A}}} d + \Sigma_{{\text{B}}} d$$

The diffusion of A into the initially pure B causes liquid swelling. In turn, both molar concentrations *c*_A_ = *n*_A_/*V* and *c*_B_ depend on time and the spatial coordinates (level coordinate *z*, distance from axis *r*), while the path length (*d*), Avogadro number (*N*_0_), and cross-sectional areas (σ) are constants; the latter were adjusted based on the observation of the pure components at the conditions of the experiment, yielding ${\Sigma }_{\text{A}}^{0}$ and ${\Sigma }_{\text{B}}^{0}$. The phase interface was detected by searching extrema of *Σ*, thus providing the interface shape and volume of the (liquid) body of revolution. Clearly, the linear attenuation coefficient of the dissolved methane can be estimated by the use of Eq. (1) assuming constant concentration of B in the liquid body corresponding to ${\Sigma }_{\text{B}}^{0}$. Its integral mean with respect to the liquid height $z \in \langle 0, Z \rangle$ was used as an accessible variable determining the liquid swelling.

2 $$\overline{{\Sigma_{{\text{A}}}^{ } }} \left( r \right) = \frac{1}{Z}\mathop \smallint \limits_{0}^{Z} \Sigma \left( {r,z} \right) - \Sigma_{{\text{B}}}^{0} {\text{d}}z = \frac{1}{Z}\mathop \smallint \limits_{0}^{Z} \Sigma_{{\text{A}}}^{{{\text{approx}}}} \left( {r,z} \right){\text{d}}z$$

The interface shape changed rapidly upon the pressure step and then remained constant to within the experimental sensitivity, while the interface position changed over time (see below). The convenient measure of swelling is:

3 $$\frac{Z}{{Z^{0} }}\left( r \right) = 1 + k \cdot \overline{{\Sigma_{A}^{ } }} \left( r \right)$$

in which *k* is radius-independent adjustable parameter and ${Z}^{0}$ is the liquid level short after the pressurization. The estimate of the distributed linear attenuation coefficient of B is then:

4 $$\Sigma_{{\text{B}}} \left( {r,z} \right) \cong \frac{{\Sigma_{{\text{B}}}^{0} }}{{1 + k \cdot \Sigma_{{\text{A}}}^{{{\text{approx}}}} \left( {r,z} \right)}}$$

The true linear attenuation coefficient of methane (*Σ*_A_) was then calculated from the overall according to Eq. (1). The use of Eq. (1) to Eq. (4) allowed for the calculation of the concentrations *c*_A_ and *c*_B_ in the liquid phase for the tomographic reconstructions, and the transformation of the physical depth coordinate (*z*) to the B-fixed coordinate $\xi \in \left\langle {0,Z^{0} } \right\rangle$, for which the diffusivity of B holds ${D}_{\text{B}}^{\text{B}}=0$. In the B-fixed reference frame, the height of the physical pixel, Δ*z*, scales to:

5 $$\Delta \xi \cong \frac{\Delta z}{{1 + k \cdot \Sigma_{{\text{A}}}^{{{\text{approx}}}} \left( {r,z} \right)}}$$

This choice of reference frame is useful for modeling diffusion in swelling bodies^32^. The Fick’s second law for axially-symmetric body in the cylindrical B-fixed coordinates then has the form^33,34^:

6 $$\frac{\partial C}{{\partial \tau }} = D_{{\text{A}}}^{{\text{B}}} \frac{{\partial^{2} C}}{{\partial \xi^{2} }} + D_{{\text{A}}}^{{\text{B}}} \frac{1}{r}\frac{\partial }{\partial r}\left( {r\frac{\partial C}{{\partial r}}} \right)$$

The above equation is a model of the concentration distribution at the central plane of the probe liquid body in the B-fixed reference frame, ${D}_{\text{A}}^{\text{B}}$ is methane (A) diffusivity in the B-fixed reference frame, and *C* = ${c}_{\text{A}}$/${c}_{\text{B}}$ = ${x}_{\text{A}}$/${x}_{\text{B}}$. We remind that ${D}_{\text{A}}^{\text{B}}$ simplifies, for instance, to the diffusivity of A in the cell reference frame (*D*_A_) if swelling and the diffusivity of B are negligible, such as for diffusion of diluted A in solid B. In this work, concentration independence of ${D}_{\text{A}}^{\text{B}}$ was assumed, and Eq. (6) was solved at the Dirichlet boundary condition (concentration at the interface set to* C*^IF^that was determined by extrapolation of concentration profiles to the phase interface) and Neumann boundary conditions (impermeable walls of the cell) using an explicit differentiation scheme^33,35^. The optimum value and uncertainty due to random errors (*u*_r_, cover factor 2) of ${D}_{\text{A}}^{\text{B}}$ and *C*^IF^were calculated using Gauss–Newton and Bonferroni methods^35,36^. The so calculated ${D}_{\text{A}}^{\text{B}}$is then the integral mean for the concentration-dependent diffusivity^32^. Importantly, molecular simulations enable the prediction of volume fraction of the species (*ϕ*_*i*_) and diffusivity in the cell reference frame (${D}_{i}$). The relation among the integral mean ${D}_{\text{A}}^{\text{B}}$ and ${D}_{i}$^32,37^ allowing for the comparison of experimental and simulated data is:

7 $$D_{{\text{A}}}^{{\text{B}}} = \frac{1}{{C^{{{\text{IF}}}} }}\mathop \smallint \limits_{0}^{{C^{{{\text{IF}}}} }} \phi_{{\text{B}}}^{2} \left[ {\phi_{{\text{A}}} \left( {D_{{\text{B}}} - D_{{\text{A}}} } \right) + D_{{\text{A}}} } \right]{\text{d}}C$$

Concentration at the phase interface was expressed using the apparent Henry’s law constant (*H*) relating methane pressure (*p*_A_) in the gas (or supercritical fluid) and its molar fraction (*x*_A_) in the liquid:

8 $$H = \frac{{p_{{\text{A}}}^{{{\text{gas}}/{\text{fluid}}}} }}{{x_{{\text{A}}}^{{{\text{liq}}}} }}$$

We remind that true Henry’s law constant is defined for the infinite dilution of A, and denoted below as $H^{\infty }$. Besides that, methane fugacity rather than pressure and the Poynting correction are generally to be used at high pressures^26^. The above simplified form of Henry’s law, Eq. (8), is practical, as it contains quantities accessible both experimentally and using molecular simulation (see below) without further conversions.

Density of the liquid at the phase interface was calculated based on the methane concentration at the interface and its partial molar volume. The latter was calculated as follows. The total amount of B in the liquid body of revolution (*n*_B,t_) was set equal to that of the initial pure B due to its small volatility^21,24^. The total amount of A in the liquid body of revolution (*n*_A,t_) was calculated based on the central-plane reconstruction using Eq. (1), while the total liquid volume takes the form according to the Euler’s first theorem for homogenous functions:

9 $$V = \overline{V}_{{\text{A}}} n_{{{\text{A}},{\text{t}}}} + \overline{V}_{{\text{B}}} n_{{{\text{B}},{\text{t}}}}$$

Apparent molar volume of methane in the liquid (${V}_{\text{A}}^{\text{app}}$) was calculated by setting ${\overline{V}}_{\text{B}}$equal to the molar volume of the pure B at the system pressure^38^. This quantity equals partial molar volume of A at infinite dilution of A, see Eq. (16).

The shape of the phase interface in the test tube in gravity possesses axial symmetry and its shape at the central plane is described by the solution of the Young–Laplace equation^39,40^:

10 $$z = \frac{\gamma }{\Delta \rho g}\left( {\frac{z^{\prime\prime}}{{\left( {1 + z^{{\prime}{2}} } \right)^{3/2} }} + \frac{z^{\prime}}{{r\left( {1 + z^{{\prime}{2}} } \right)^{1/2} }}} \right)$$

Equation (10) can be numerically solved for *z'*(*r* = 0) = 0 and *z'*(*r* = *R*) = cot(*θ*), the distance form axis ranges from zero to the tube inner radius, *r* $\in$ (0, *R*). Parameters have the usual meaning: density difference at the interface (Δ*ρ*), interfacial energy (*γ*), contact angle (*θ*). Density of the gas phase (methane) was calculated using the Setzmann–Wagner equation of state^41^. Density of the liquid at the interface was calculated from the respective concentrations and molar volumes at the interface as described above.

### Molecular dynamics

MD simulations were used to model the macroscopic behavior of experimentally investigated 2-phase systems from methane (A) + *p*-xylene (B), exceed the range of experimentally achieved conditions in this work, and gain microscopic insight into the structure of bulk phases and of the interface. MD simulations were performed with united-atom Trappe force-field^42^, which is an efficient simulation model due to its simplicity (united atom, no partial charges), transferability, and very good accuracy owing to benchmarking to gas–liquid equilibrium experimental data. Lennard–Jones (LJ) cut-off was set to 2.99 nm, which was shown to quantitatively reproduce the phase behavior and also surface tension (Fig. S1 in Supplementary Information, SI) of neat *p*-xylene. All MD simulations were performed in the GROMACS simulation package^43^ with a timestep of 2 fs. The simulated systems were divided into two main groups by the number of phases in the system.

**Fig. 2:**
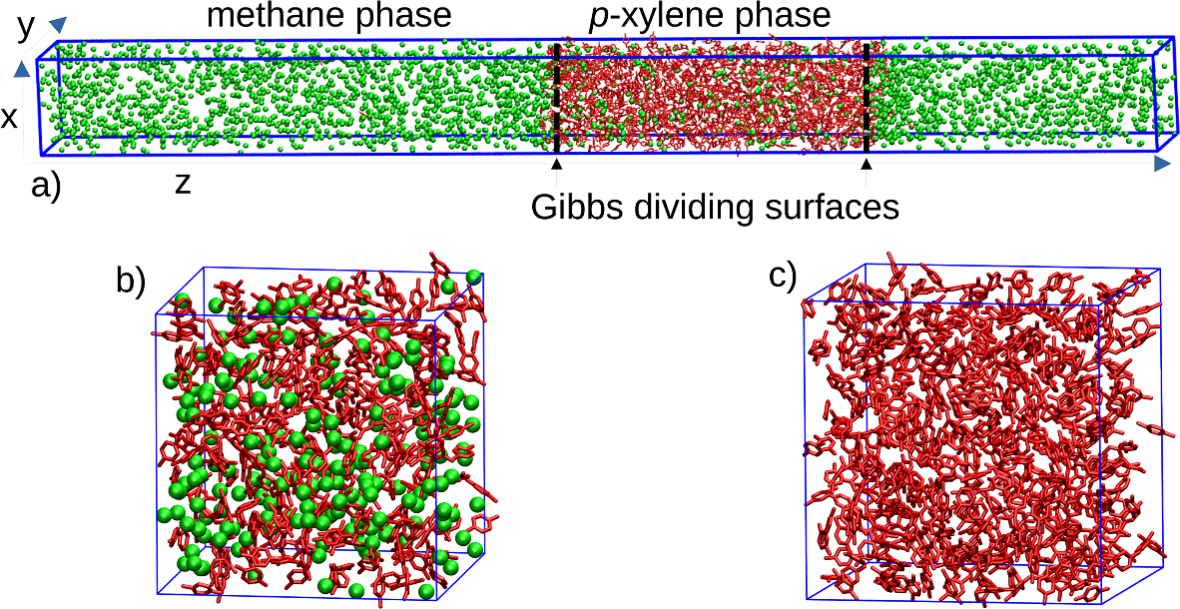
Simulation setups used in this work to determine target macroscopic properties, and to get insight in the structure of the solution and of the interface. **a** slab simulation setup, **b** homogeneous phase of *p*-xylene with dissolved methane, **c** pure *p*-xylene phase. Methane is in green spheres, *p*-xylene in red licorice.

First, we have simulated systems containing 2 coexisting phases with an explicit presence of the interface, *i.e*. the slab simulation setup (Fig. 2a). This setup enabled the evaluation of surface tensions, equilibrium density profiles across the interface (Fig. S2 and Fig. S3 in SI), and the direct measurement of the apparent Henry’s law constants. The system for the slab simulations was a rectangular cuboid with side lengths of 6.0 nm, 6.0 nm and 50 nm. System consisted of 1000 *p*-xylene molecules and 400–5000 methane molecules, which were distributed in the *p*-xylene-rich liquid phase (*x*_A_^liq^= 0 to 0.25) and in the methane gas phase (according to experiment and Henry’s law) utilizing the PACKMOL package^44^. The slab was equilibrated for 10 ns in semi-isobaric NpT ensemble (compressible in *z*-direction only) in order to obtain targeted pressure. The production was carried out in NVT ensemble with the total simulation time 40 ns from which the first 20 ns were used as equilibration to ensure that equilibrium of methane between gas and *p*-xylene liquid phase is reached (via convergence of density profiles). All 2-phase simulations were performed with two separate V-rescale thermostats^45^ (τ_*T*_ = 0.1 ps) used for temperature coupling of methane and *p*-xylene.

Second, we have carried out bulk simulations (Fig. 2b, c), which present an efficient and reliable route for the determination of bulk properties, such as molar volume, diffusion, viscosity, density, true Henry’s law constant, chemical potentials or local solution structure (Fig. S4 in SI). These simulations were performed in isobaric-isothermal (NpT) ensemble with C-rescale barostat (τ_*p*_ = 2 ps) and V-rescale thermostat (τ_*T*_ = 0.1 ps). Simulation time was 40 ns with the first 20 ns used for equilibration and not used for analysis. The system consisted of 1000 particles, the numbers of methane and *p*-xylene particles were varied.

### Theory

Surface tension was calculated from the pressure tensor, which was measured during the slab simulations (Fig. 2a) using the following equation:

11 $$\gamma \left( t \right) = \frac{{L_{z} }}{2}\left( {P_{zz} \left( t \right) - \frac{{P_{xx} \left( t \right) + P_{yy} \left( t \right)}}{2}} \right)$$

where *L*_*z*_ is the length of the simulation box along the *z*-axis, *P*_*zz*_ is the perpendicular component (and the macroscopic pressure in the system), while *P*_*xx*_, *P*_*yy*_ are the lateral components.

The diffusion coefficient of methane in the *p*-xylene solution was calculated from the mean square displacement according to Einstein’s formula (*D*_PBC_, Eq. (12), literature^46^). In order to account for long range hydrodynamic effects due to PBC in finite systems, finite size correction was applied and system size independent (true) *D*_0_ evaluated via Eq. (13) as recommended in the literature^11^. In Eq. (13), *k*_B_ and *T* are the Boltzmann constant and thermodynamic temperature, constant *ξ*= 2.837297^46^, *η*_MD_ is *p*-xylene viscosity, and *L* is the side of the cubic simulation box.

As diffusivity is inversely proportional to the viscosity of the solvent medium, another correction [*η*_MD_/*η*_exp_ in Eq. (14)] is routinely applied in the literature, accounting for the difference between pure solvent (*p*-xylene) viscosity in the simulation (*η*_MD_) and in the experiment (*η*_exp_). This factor was determined based on experimental and MD viscosity data at 293 K^47^. This uniform scaling proved quantitative for solutions, the viscosity of which does not significantly vary with composition (*e.g*. pure liquids, diluted solutions). However, in the case of complex solutions mixtures of significantly varying density (10–20 mol.% of supercritical methane in liquid *p*-xylene) for which experimental viscosity data are not available, the scaling based on pure liquid viscosities cannot be expected to be quantitative. To practically overcome these various limitations and uncertainties, we have introduced a universal effective scaling parameter [*k*_*η*_ in Eq. (14)] by calibrating diffusion coefficients from MD simulations to our experimental data. We note that scaling performed in Eq. (14) does not bias temperature or composition dependences of diffusion coefficients (A, B). Moreover, *k*_*η*_ is not expected to significantly deviate from unity, and *k*_*η*_ = 1 in pure liquids (and at infinite dilution of solute).

12 $$D_{{{\text{PBC}}}} = \frac{1}{6}\mathop {\lim }\limits_{t \to \infty } \frac{\partial }{\partial t}{\text{MSD}}\left( t \right)$$

13 $$D_{0} = D_{{{\text{PBC}}}} + \frac{{k_{{\text{B}}} T\xi }}{{6\pi \eta_{{{\text{MD}}}} L}}$$

14 $$D = D_{0} \frac{{\eta_{{{\text{MD}}}} }}{{\eta_{{{\text{exp}}}} }}k_{\eta }$$

Solution viscosity is another important property, which steps in the continuous modeling and interpretation of time-resolved data of neutron imaging. In this theoretical study, we have calculated viscosity via Einstein’s approach (-evisco option in gmx energy routine of GROMACS package). To improve convergence, the averaging was performed over 100 independent simulations, each of a length of 2 ns, following the recommendation from the literature^48^.

The volumetric properties, namely partial molar volumes (${\overline{V}}_{i}$) and volume fractions (${\phi }_{\text{i}}$), present an important input to continuous modeling. Their evaluation from MD simulations starts by a direct calculation of apparent molar volume of methane (${V}_{\text{A}}^{\text{app}}$) according to Eq. (15), which requires only system volumes for a series of compositions, *i.e*., numbers of molecules [$V\left({N}_{\text{A}},{N}_{\text{B}}\right)$] and that of pure *p*-xylene liquid ($V_{{\text{B}}}^{ \bullet }$)^13^.

15 $$V_{{\text{A}}}^{{{\text{app}}}} = \frac{{N_{0} }}{{N_{{\text{A}}} }}\left( {V\left( {N_{{\text{B}}} ,N_{A} } \right) - V_{{\text{B}}}^{ \bullet } N_{{\text{B}}} } \right)$$

Following the formula from the literature^49^, partial molar volume of methane (${\overline{V}}_{\text{A}}$) is determined according to Eq. (16)

16 $$\overline{V}_{{\text{A}}} = V_{{\text{A}}}^{{{\text{app}}}} + x_{{\text{A}}} x_{{\text{B}}} \left( {\frac{{\partial V_{{\text{A}}}^{{{\text{app}}}} }}{{\partial x_{{\text{A}}} }}} \right)$$

In case that ${V}_{\text{A}}^{\text{app}}$ is linear in ${x}_{\text{A}}$, *i.e*. ${V}_{\text{A}}^{\text{app}}= {V}_{\text{A}}^{\infty }+b{x}_{\text{A}}$, the partial molar volume of methane takes a simple form ${\overline{V}}_{\text{A}}= {V}_{\text{A}}^{\text{app}}+b{x}_{\text{A}}{x}_{\text{B}}={V}_{\text{A}}^{\infty }+b{x}_{\text{A}}(1+{x}_{\text{B}})$. The calculation of ${\overline{V}}_{\text{B}}$ is straightforward. The molar fractions and partial molar volume of *p*-xylene are determined from known composition (molar concentrations, *c*_*i*_) according to:

17 $$\phi_{i} = c_{i} \overline{V}_{i}$$

Insight into the *p*-xylene-methane interactions is captured in the excess (residual) chemical potential^50^. That of methane in the *p*-xylene phase was efficiently calculated via the Widom insertion method, where *ψ* is the interaction energy of an inserted methane particle and the ensemble average $\langle .\rangle$ is performed over configurations (20 000 frames) of *p*-xylene phase. 20 000 methane insertions per frame were attempted.

18 $$\mu_{{\text{A}}}^{{{\text{ex}}}} = - k_{{\text{B}}} T{\text{ln}}\left\langle {{\text{e}}^{{ - \frac{\psi }{{k_{{\text{B}}} T}}}} } \right\rangle$$

This opens a path for a calculation of the true Henry’s law constant (low methane pressure) for methane into pure *p*-xylene liquid of particle density (molar concentration) ρ_B_

19 $$H^{\infty } = \rho_{{\text{B}}} k_{{\text{B}}} T{\text{e}}^{{\frac{{\mu_{{\text{A}}}^{{{\text{ex}}}} }}{{k_{{\text{B}}} T}}}}$$

thus independently confirming the equilibrium methane concentration in *p*-xylene, which was determined directly in the slab simulation. The apparent Henry’s law constant, determined at finite methane pressures, was calculated from slab simulations according to Eq. (8).

## Results and discussion

**Fig. 3:**
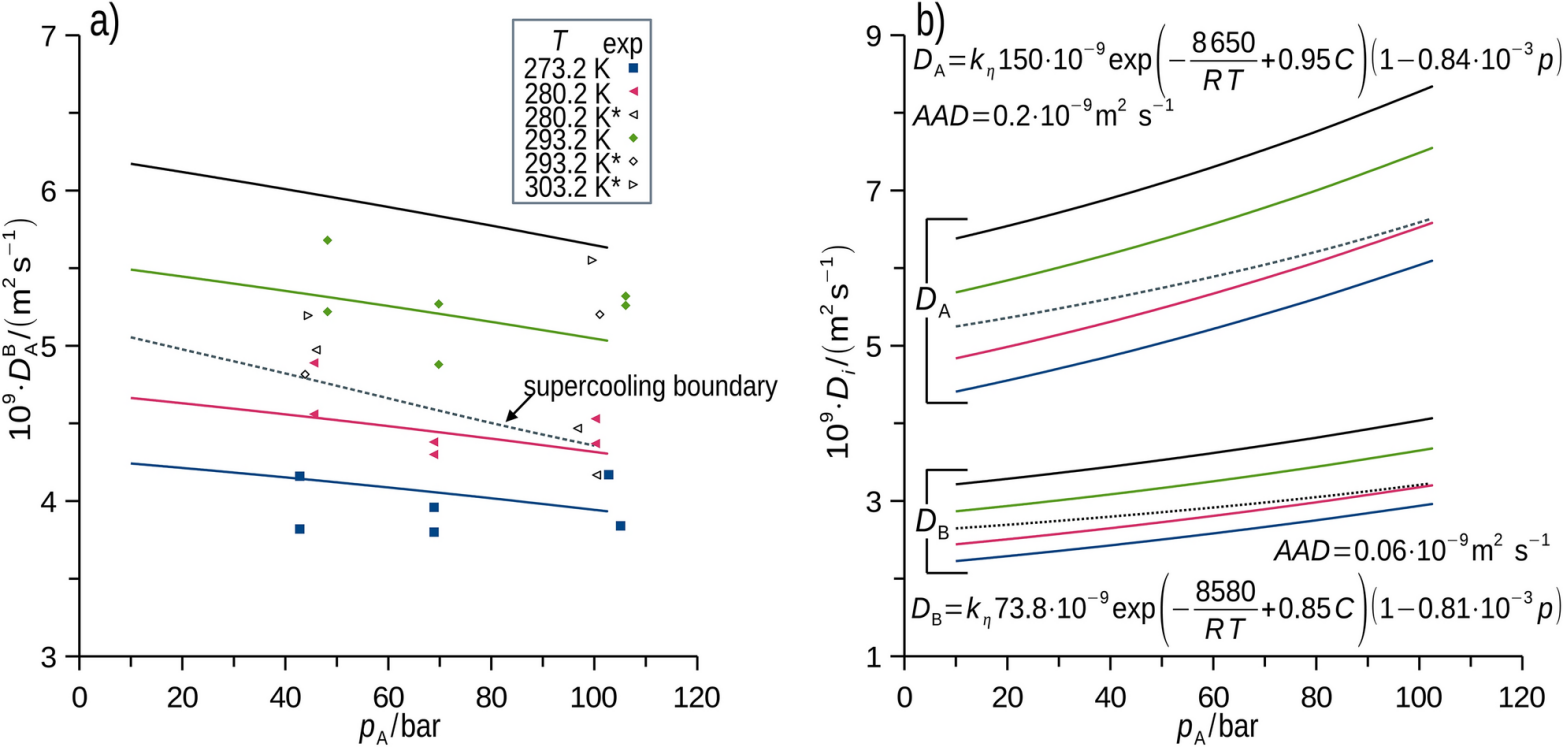
Integral mean diffusivity of methane in *p*-xylene (${D}_{\text{A}}^{\text{B}}$) for the B-fixed reference frame is shown in (**a**). Points were observed using neutron imaging, curves are regressions derived from MD simulation processed using Eq. (7), * indicates data from our previous study^7^. Regressions of the MD simulated diffusivity for A and B in the cell reference frame derived for ranges of temperature (260 to 400 K), concentration (0.01 to 0.33 mol(A)/mol(B)), absolute pressure (10 to 100 bar) are shown in (**b**). Simulated data for the conditions at which solidification occurs^4^ are shown (dashed curve, supercooling boundary). Average uncertainty of the experimental ${D}_{\text{A}}^{\text{B}}$ equals 0.2 × 10^–9^ m^2^s^-1^. Average uncertainty of simulated *D*_A_ and *D*_B_ is 0.3 × 10^–9^ m^2^s^-1^ and 0.1 × 10^–9^ m^2^s^-1^, respectively. *k*_*η*_ = 1.284 was used in processing of MD simulation data [see Eq. (14)] to effectively account for unknown experimental viscosity of complex *p*-xylene solutions saturated by methane at elevated pressures.

Experimental data derived from the neutron imaging of the supercritical methane (CH_4_, component A) absorption in liquid perdeuterated *p*-xylene (*p*-C_8_D_10_, component B), and the results of the MD simulation are compared in this section, followed by predictive simulations. Figure 3a shows the simulated mean diffusivity of A in the liquid B in the experimentally accessible B-fixed reference frame (${D}_{\text{A}}^{\text{B}}$), see Eq. (7), together with the selection of experimental results. Complete sets of experimental and simulated results are available in the Supplementary Dataset (SD). The supercooling boundary, that is the equilibrium condition at which solid *p*-C_8_D_10_ occurs^4,7^, is shown. No influence of the supercooling on the master trends was discerned either for the experimental or simulated diffusivity. As a calibration effort, the experimentally derived ${D}_{\text{A}}^{\text{B}}$ was predicted quantitatively using MD (Fig. 3a), which well captures trends in temperature and pressure after adjusting the viscosity scaling parameter. It is noteworthy that diffusion flux of methane through the phase interface causes accumulation at the interface^10^, which is a possible reason for the inertia of the boundary condition upon the step pressurization reported in our previous works on the *one-pot imaging*^7,8^. In contrast to the experimental setup, diffusion in MD was determined from fluctuations in an equilibrium system with no macroscopic flux. The respective diffusivities of methane (A) and *p*-xylene (B) in the binary liquid solutions in cell coordinates (*D*_*i*_) calculated using MD (Fig. 3b) are presented as regressions (models fit to data) with shown average absolute deviation (*AAD*).

**Fig. 4:**
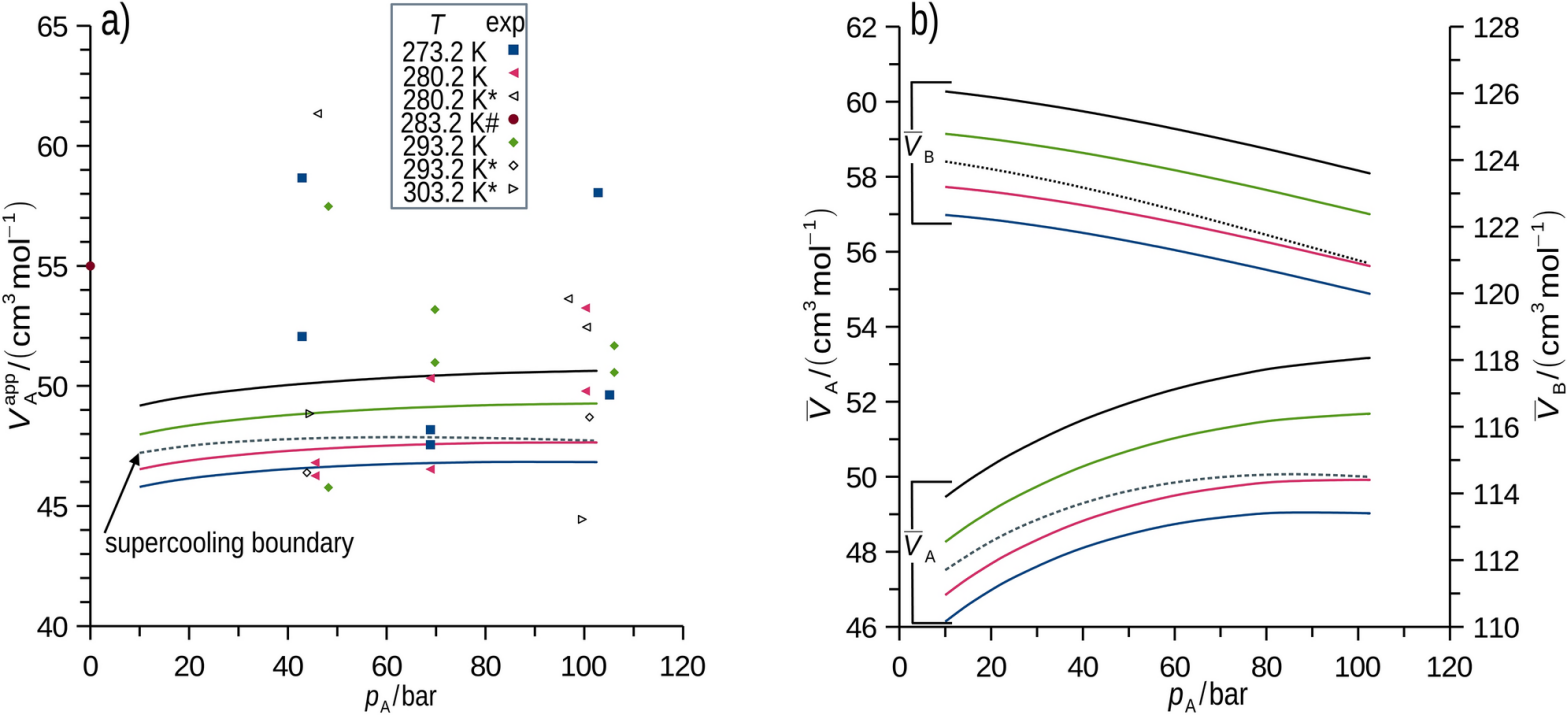
Apparent volume of methane in *p*-xylene, average experimental uncertainty is 5 cm^3^mol^-1^, upper estimate of uncertainty for simulation is 0.5 cm^3^mol^-1^ (at *x*_A_ = 0.05, decreases with increasing concentration), *AAD* for the MD simulated data regression is 0.5 cm^3^mol^-1^ (**a**). Simulated partial molar volumes of methane and *p*-xylene at the indicated conditions, *AAD* for partial molar volume regression is 0.6 cm^3^mol^-1^ and 0.2 cm^3^mol^-1^ for methane and *p*-xylene, respectively. Upper estimates of uncertainty for partial molar volume simulation is 0.5 cm^3^mol^-1^ and 0.03 cm^3^mol^-1^ for methane and *p*‑xylene, respectively (**b**). * indicates data from^7^, # indicates datum for methane and *n*-hexane from^54^.

Measured and simulated apparent molar volume of methane in *p*-xylene (${V}_{\text{A}}^{\text{app}}$) compared well within the uncertainties (selection is in Fig. 4a, measured and simulated results for all studied conditions are in SD). Thus, partial molar volumes of both components (${\overline{V} }_{i}$) were predicted using MD (Fig. 4b), see Eq. (16). Importantly, MD provided partial molar volume of the major component, B, that is not accessible using the used experimental setup. The species volume fractions (*ϕ*_*i*_) were calculated using Eq. (17).

Simulated (true) Henry’s law constant ($H^{\infty }$) for infinitely diluted methane in *p*-xylene rose slightly with the increasing absolute pressure and with increasing temperature (simulation results are in SD). The following regression, in which $H^{\infty }$ and *p* are in bar, *T* in K, and *R* = 8.31451 JK^-1^ mol^-1^^41^, approximated the results at *AAD* = 2 bar for *T* and *p* ranging 260 to 400 K and 10 to 100 bar, respectively.

20 $$H^{\infty } = 52\exp \left( {\frac{14400}{{RT}} - \frac{{3.12 \cdot 10^{6} }}{{RT^{2} }} + 161 \cdot 10^{ - 5} p} \right)$$

**Fig. 5:**
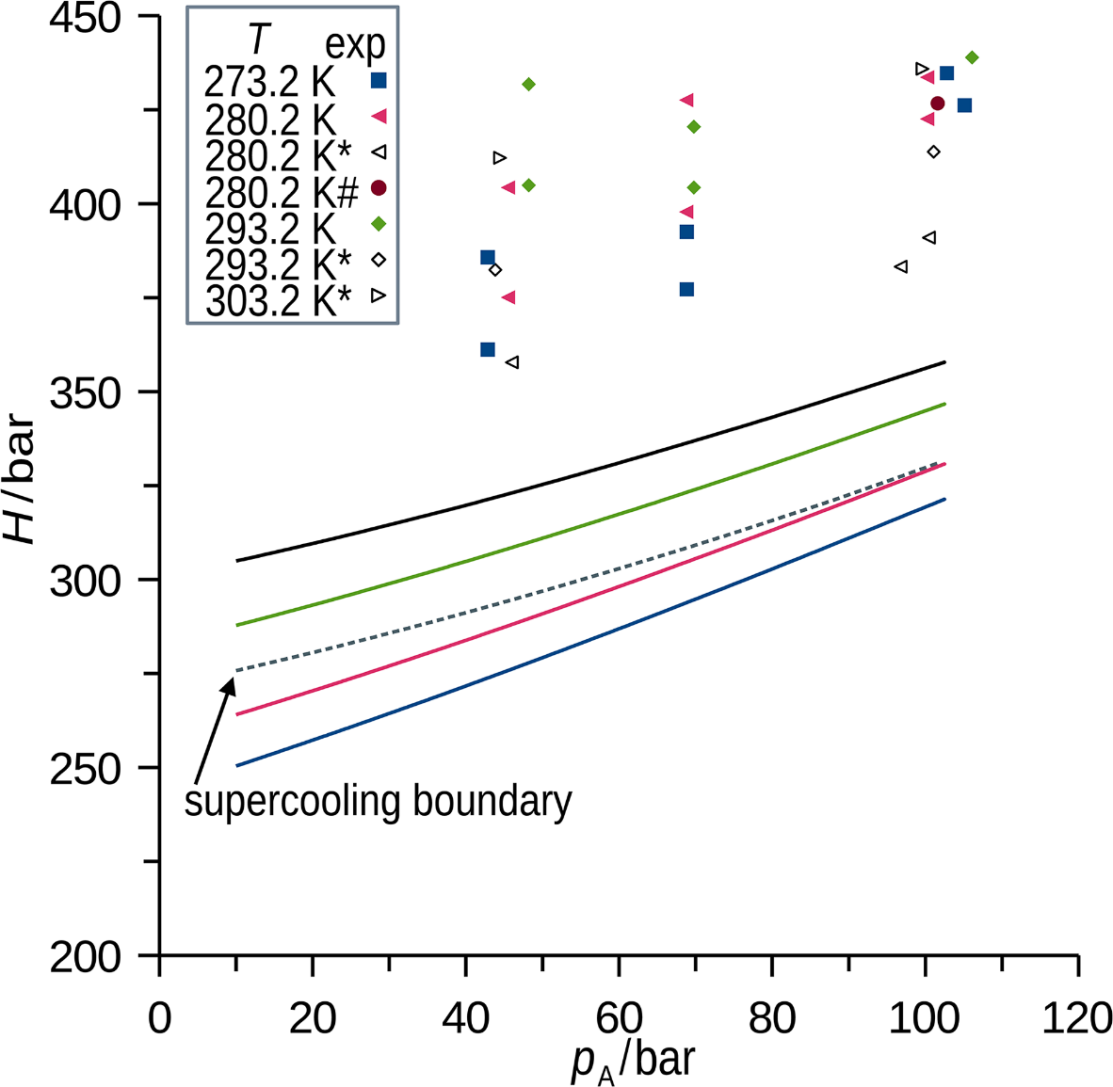
Apparent Henry’s law constant (*H*) for methane in *p*-xylene, see Eq. (8). Points were observed using neutron imaging, curves represent regression of simulated data (*AAD* = 5 bar), average relative deviation of simulation from the experiment was approximately 30%, which corresponds to the uncertainty of 1 kJ mol^-1^ in ${\mu }_{\text{A}}^{\text{ex}}$. * indicates data from^7^, # indicates datum from^4^.

The measured and simulated apparent Henry’s law constant [Eq. (8)] are compared in Fig. 5. Simulation systematically underestimated experimental apparent Henry’s law constants by 100 ± 25 bar. This originates in the exponential dependence of the Henry’s law constant on the excess chemical potential ${\mu }_{\text{A}}^{\text{ex}}$ [see Eq. (19)]. Although the force field approximated the system adequately, the error of 1 kJ mol^-1^ in ${\mu }_{\text{A}}^{\text{ex}}$ propagates as ${\text{e}}^{-\frac{\text{err}({\mu }_{\text{A}}^{\text{ex}}) }{RT}}$ and results in the error of 30% in ${H}^{\infty }$.

Since the macroscopic observables were quantitatively captured by MD simulations, the molecular insight into the solution structure of investigated solutions may follow. The structure of a bulk solution of *p*-xylene with methane is described by series of radial distribution functions (RDF) for increasing molar fraction of dissolved methane. For illustration, Fig. S4 (SI) presents mutual distributions of methane and *p*-xylene molecules and associated running coordination numbers. Only minor changes in RDFs with methane concentration are found, suggesting that methane dissolves well in *p*-xylene and together form nearly regular solution.

The quantitative insight into composition of the interfacial region is captured in *z*-resolved density profiles of *p*-xylene and methane. Fig. S2 (SI) illustrates the composition for a series of methane pressures (at 298 K). It is confirmed that methane density in the *p*-xylene phase is lower than in the gas phase, methane is surface active, and its surface excess relatively decreases with increasing methane pressure. A random simulation snapshot at 298 K, 45 bar (Fig. S3, SI) presents the side and top view to the structure and arrangement of the intrinsic interface. Qualitatively, the surface excess of methane and lowering of methane concentration in the *p*-xylene phase is visually observed. Importantly, these snapshots confirmed that the methane distribution within any of the interfacial layers is random, *i.e*., no methane-rich associates or domains are formed.

**Fig. 6:**
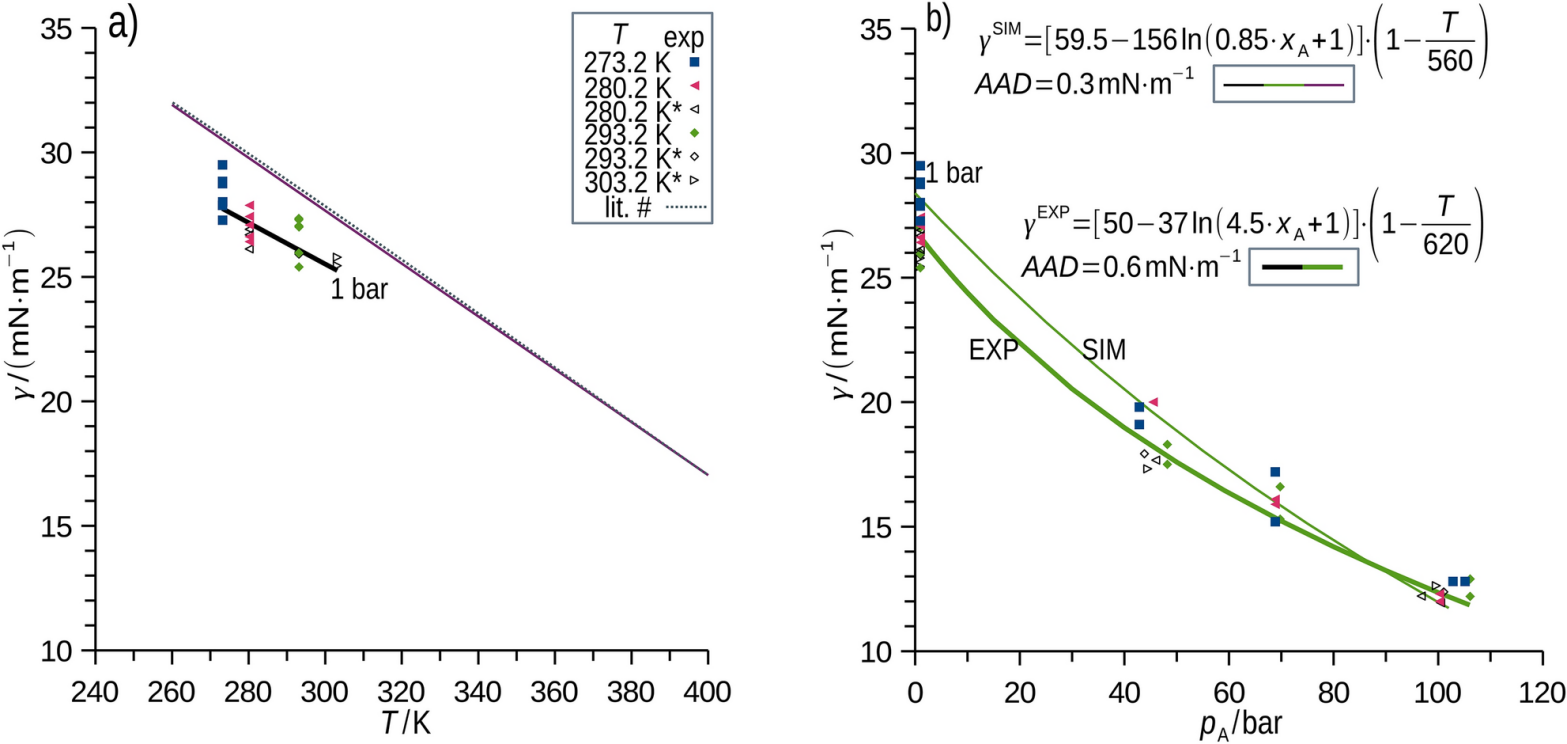
Surface energy observed experimentally using neutron imaging and simulated, regression equations are shown in (**b**), their plots for 1 bar and 293.2 K are in (**a**) and (**b**), respectively. Regression of the MD simulated interfacial energy was derived for broad ranges of *T* (260 to 400 K) and *x*_A_ (0 to 0.3). Regression of the experimental interfacial energy was derived for ranges of *T* (273.2 to 303.2 K) and *x*_A_(0 to 0.26). Comparison to the literature data^7,51^ (* and #) is shown in (**a**).

Measured surface energy at 1 bar (methane, liquid *p*-xylene) is shown for selected conditions in Fig. 6a (all data are in SD). While simulation effectively resembled the literature data for the pure *p*-xylene^7,51^, systematic error of approx. 2 mN m^-1^ was observed for the experimental data at 1 bar, for which the average (random) uncertainty of the interfacial energy measurement was 2 mN m^-1^. The influences of the cell alignment with respect to gravity and the presence of methane at 1 bar were checked by measuring with tilted apparatus (± 1°) and upon removal of methane (vacuuming) without observing systematic changes of surface energy and its uncertainty. Simulated and measured surface energy for the interface of methane and *p*-xylene showed similar trends, while the systematic experimental error (2 mN m^-1^) vanished with increasing pressure (Fig. 6b). The average uncertainty of the interfacial energy measurement at elevated pressures is 1 mN m^-1^, attributed to the higher sensitivity of the method at lower surface energies^52^. MD simulation resembled experimental interfacial energies within the achieved uncertainties. This fully justifies the use of MD for predictions within the experimentally provided temperature and pressure domain, and supports its use for the parameter domains outside those calibrated by the provided experiments.

**Fig. 7:**
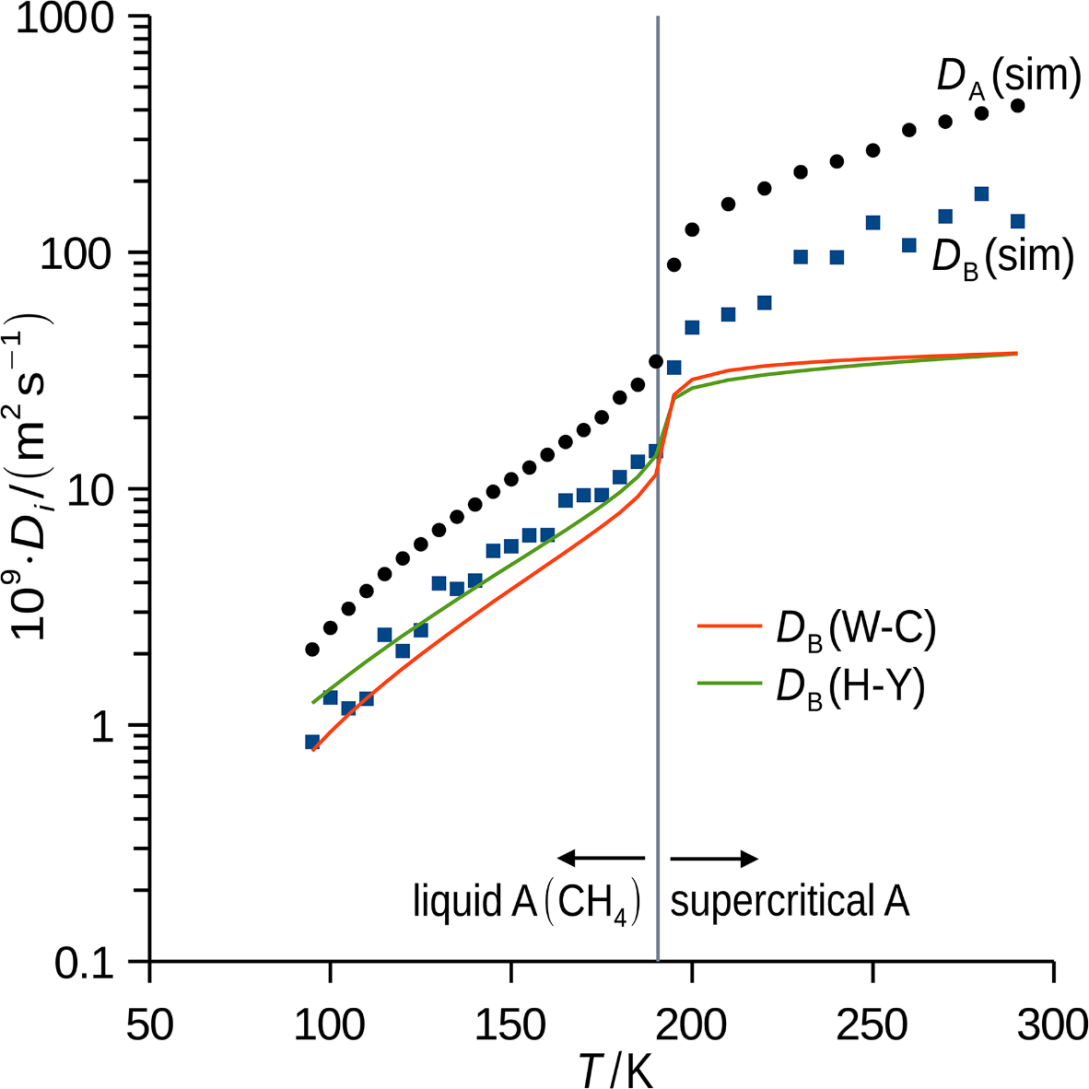
Simulated diffusivity of *p*-xylene (component B) and methane (component A) at the infinite dilution of B at 50 bar, curves are engineering predictions according to Wilke and Chang (W–C)^25^, and He and Yu (H–Y)^27^. Fluid properties of pure methane were taken from database^53^, constants from database^51^. *k*_*η*_ = 1 was used in processing of MD simulation data [see Eq. (14)] as *p*-xylene is present at infinite dilution.

Simulated diffusivities of *p*-xylene and methane at the infinite dilution of *p*-xylene at 50 bar followed expectable trends (Fig. 7) within the studied conditions that correspond to liquid and supercritical methane^41^. Diffusivity of infinitely diluted *p*-xylene was also predicted using the engineering equations according to Wilke and Chang^25,26^, and He and Yu^26,27^ with parameters from the databases^51,53^. These predictions differed, on average, by just 28% (Wilke and Chang), 17% (He and Yu), and 11% (Wilke and Chang with association factor adjusted to 1.963, curve not shown in Fig. 7﻿) from the simulated *p*-xylene diffusivity in its infinitely diluted solution in liquid methane. On the contrary, substantial differences (average 65% of the simulated value for both equations) were observed for the supercritical methane at reduced density down to 0.2. It is noteworthy that the more recent He and Yu^26,27^ model was developed mainly based on data for systems of higher reduced densities. As a result, a high diffusion flux, and consequently intensive solid *p*-xylene deposition at cold spots during the supercritical methane cooling, is predicted. Moreover, MD enabled the prediction of the self-diffusivity of the major component (methane) at the infinite dilution of *p*-xylene, at the same conditions.

## Conclusion

This study provides novel experimental and molecular dynamics insights into the properties of the two-phase methane and liquid *p*-xylene systems, which is an industrially relevant pair for natural gas processing. Through the combination of neutron imaging and MD simulations, key properties such as methane diffusivity, Henry’s law constant, apparent molar volume, and surface tension were determined and compared. The results demonstrate that MD simulations align with experimental data with differences within acceptable limits, thereby validating the MD model under the studied conditions. While the experimental study was conducted at temperature and pressure ranging 0.0 to 20.0 °C and 1 to 100 bar, respectively, MD enabled the prediction of the system properties for a broad range of temperatures (260–400 K) at pressures up to 100 bar.

MD simulations allowed for predictions of system properties at experimentally inaccessible conditions. The prominent example is the prediction of *p*-xylene diffusivity in liquid and subsequently in supercritical methane (both done using infinitely diluted *p*-xylene), with the latter found significantly higher than that predicted using common engineering correlations (Wilke–Chang and He–﻿Yu). These MD simulations thus predict intensive freeze-out formation, and shed light on the understanding of the behavior of volatile impurities in natural gas, which is related to operational challenges in natural gas liquefaction.

In conclusion, the integrated experimental and computational approach adopted here enables a deeper understanding of the methane-*p*-xylene system, providing valuable data for natural gas industries and establishing a foundation for further exploration of complex fluid systems. Building on these findings, we will apply similar experimental and simulation protocols to other industrially relevant systems.

## Supplementary Information

Supplementary Information.

Supplementary Dataset.

## Data Availability

Experimental data will be made available upon request, additional data are provided in Supplementary Dataset. Inputs for molecular dynamics simulations and raw simulation data for selected systems are available via Zenodo: https://doi.org/10.5281/zenodo.14266923.
