# Supplementary material for: Neutron imaging and molecular simulation of systems from methane and p-xylene
Source: Sci Rep. 2025 Jan 8;15:1284. doi: 10.1038/s41598-024-85093-6 (PMC11711756; doi:10.1038/s41598-024-85093-6)
Supplement: Supplementary file 1 — Supplementary Information. [file 41598_2024_85093_MOESM1_ESM.docx]

Supplementary Information

**Neutron imaging and molecular simulation of systems from methane and *p*‑xylene**

*Martin Melčák^1†^ Tereza-Markéta Durďáková^1†^, Štěpán Tvrdý^1^, Jonatan Šercl^1^, Jong Min Lee^2^, Pierre Boillat^2,3^, Jan Heyda^1^, Pavel Trtik^2,*^, Ondřej Vopička^1,*^*

^1^ Department of Physical Chemistry, University of Chemistry and Technology, Prague, Technická 5, 166 28 Prague 6, Czech Republic

^2^ Laboratory for Neutron Scattering and Imaging, Paul Scherrer Institut, 5232 Villigen PSI, Switzerland

^3^ Electrochemistry Laboratory, Paul Scherrer Institut, 5232 Villigen PSI, Switzerland

^*^ Corresponding authors: [ondrej.vopicka@vscht.cz](mailto:ondrej.vopicka@vscht.cz), [pavel.trtik@psi.ch](mailto:pavel.trtik@psi.ch)

*^†^* Authors contributed equally

**DETAILS ON MOLECULAR DYNAMICS CALCULATIONS**

The Validation of the simulation setup (cutoff, system size) based on a comparison of simulated and experimental surface tension is shown in Figure S1. Density profiles (quantitatively analyzed) at the interface are presented in Figure S2, and the particle distribution in the interfacial region are illustrated in Figure S3. The solution structure in the homogeneous *p*-xylene with dissolved methane and its changes with methane fraction are provided in Figure S4.

Figure S1 presents the effect of the short-range interaction (Lennard-Jones) cut-off on surface tension. A systematic offset by 1-2 mN/m is related to the explicit accounting of these interactions. The comparison with literature-reported experimental data led to the choice of system sizes (6x6x50 nm) used in this work.

Figure S2 illustrates the (time/ensemble) averaged *z*-density profiles (for methane and *p*-xylene) for *T* = 298 K. Several representations are shown to illustrate mass, particle and relative distribution of the two species at the interface as well as in the bulk phases.

Figure S3 shows the intrinsic structure from a selected system (*T* = 298 K, *p*_methane_ = 45 bar, green lines in Figure S2), visualizing the methane particles in selected layers parallel to the interface.

Figure S4 presents changes in the solution structure (radial distribution functions (RDF)) with methane concentration (from 1 to 20 mol.% of methane) in the *p*-xylene phase. Intermediate conditions were selected, *i.e*., *T* = 298 K and external pressure of 45 bar. Results show the particle distribution around methane and *p*-xylene molecules, as well as associated running coordination numbers. The observation that changes in RDFs with methane concentration are minor proves that methane in *p*-xylene forms a nearly regular solution.


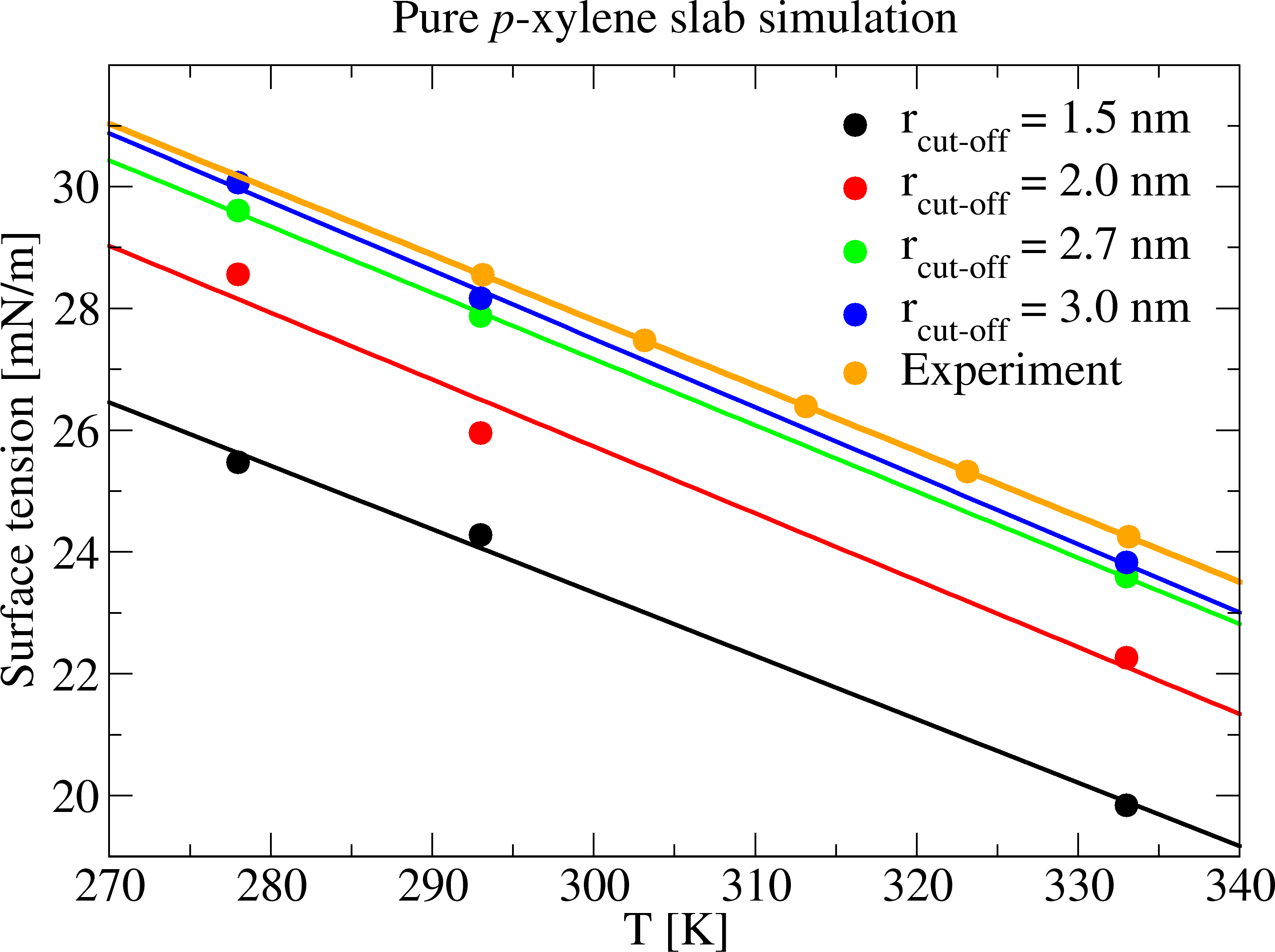


Figure S1. Dependence of the neat *p*-xylene surface tension obtained from MD simulations with a united atom TraPPE force-field (points and linear regression fits) on the applied cut-off distance of Lennard-Jones potential (see the legend). The experimental data from the literature ^1^ (orange) are shown for reference. The *x*,*y*-dimensions of the simulation box in this work were set to 2×3 = 6 nm, which was also the thickness of the *p*-xylene phase.


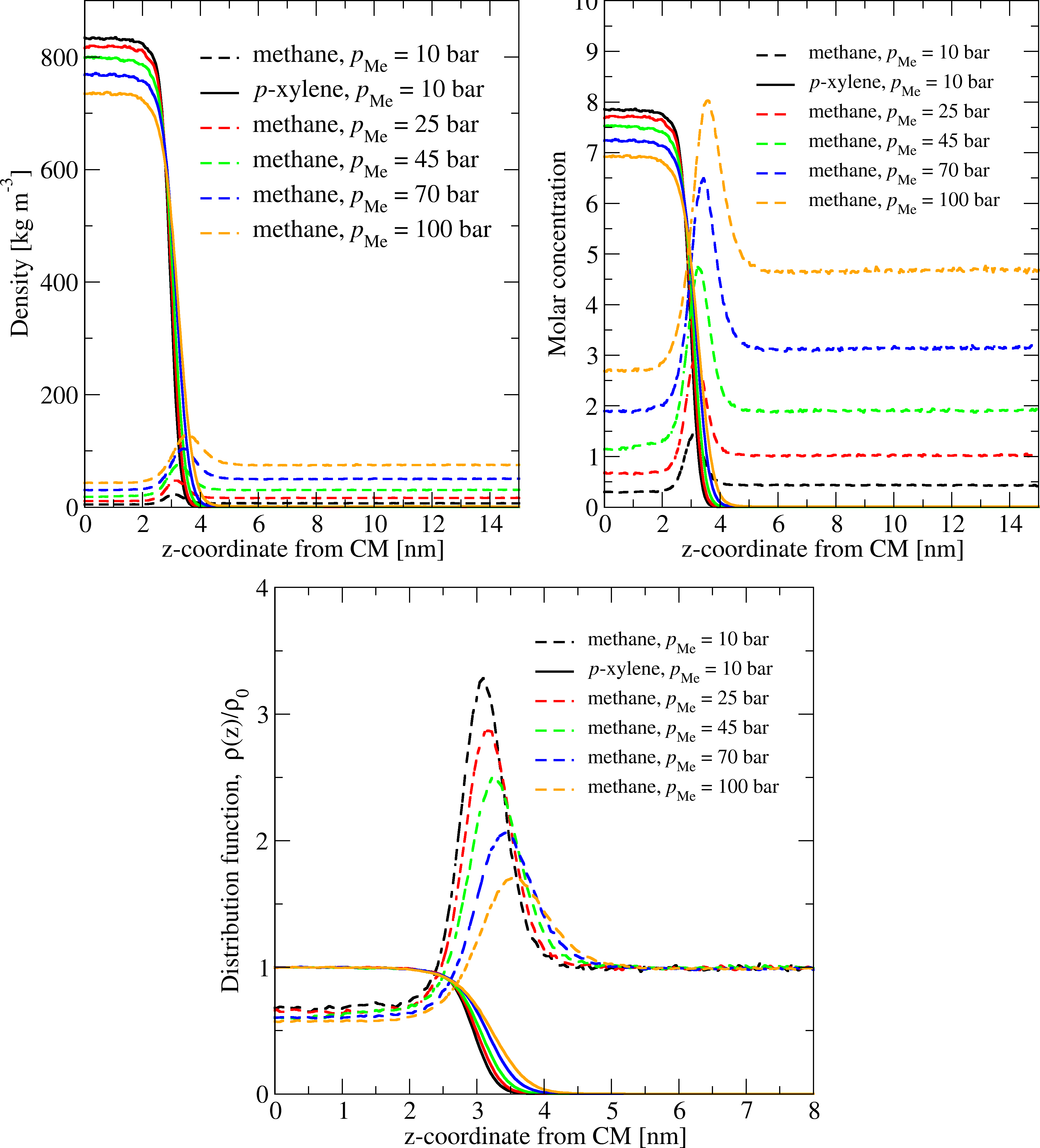
Figure S2. Average density profiles resolved in the normal direction (z-axis) relative to the interface were determined for *p*-xylene/methane systems at 298 K and methane pressure of 10-100 bar. The top panel presents the *p*‑xylene (full line) and methane (dashed line) density in mass (left) and molar (right) scales, respectively. The bottom panel presents the distribution functions (normalized to the bulk densities of individual species). A broadening of the *p*-xylene interface (from ca 1.0 nm to ca 1.5 nm) and a reduction in the relative surface excess of methane (from ca 3.5× to 1.7×) with increasing methane pressure (from 10 bar to 100 bar) are clearly visible.


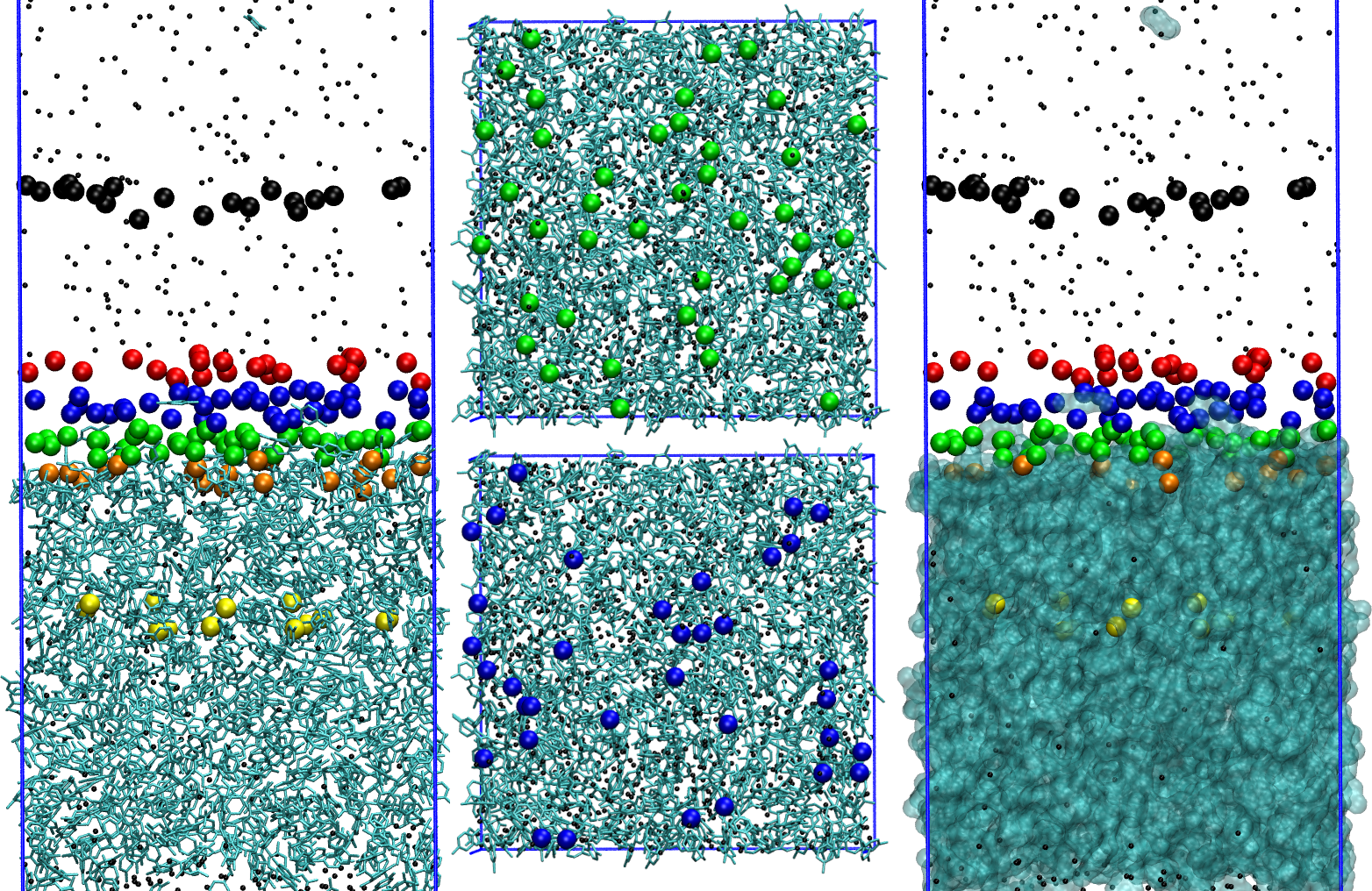
Figure S3. An illustrative snapshot of the intrinsic structure of methane (black spheres representation) and *p*-xylene (cyan licorice) molecules at the interface region taken at 298 K and 45 bar of methane. The side view (left) of the simulation box shows methane (at 45 bar) in contact with *p*-xylene phase. Methane particles at selected 5Å-thick layers are shown using large vdW sphere representation, so that the surface enrichment of methane (ca 2x compared to the gas phase) and the reduced concentration of methane in the *p*-xylene phase (ca 0.6x that of the gas phase) are illustrated. This is consistent with the *z*-density profiles in Figure S2 (dashed green lines). The top view of the interface (right) shows that the methane distribution at the interface (green), and above the *p*‑xylene phase (blue) is random, *i.e*., no methane-rich associates or domains are formed.


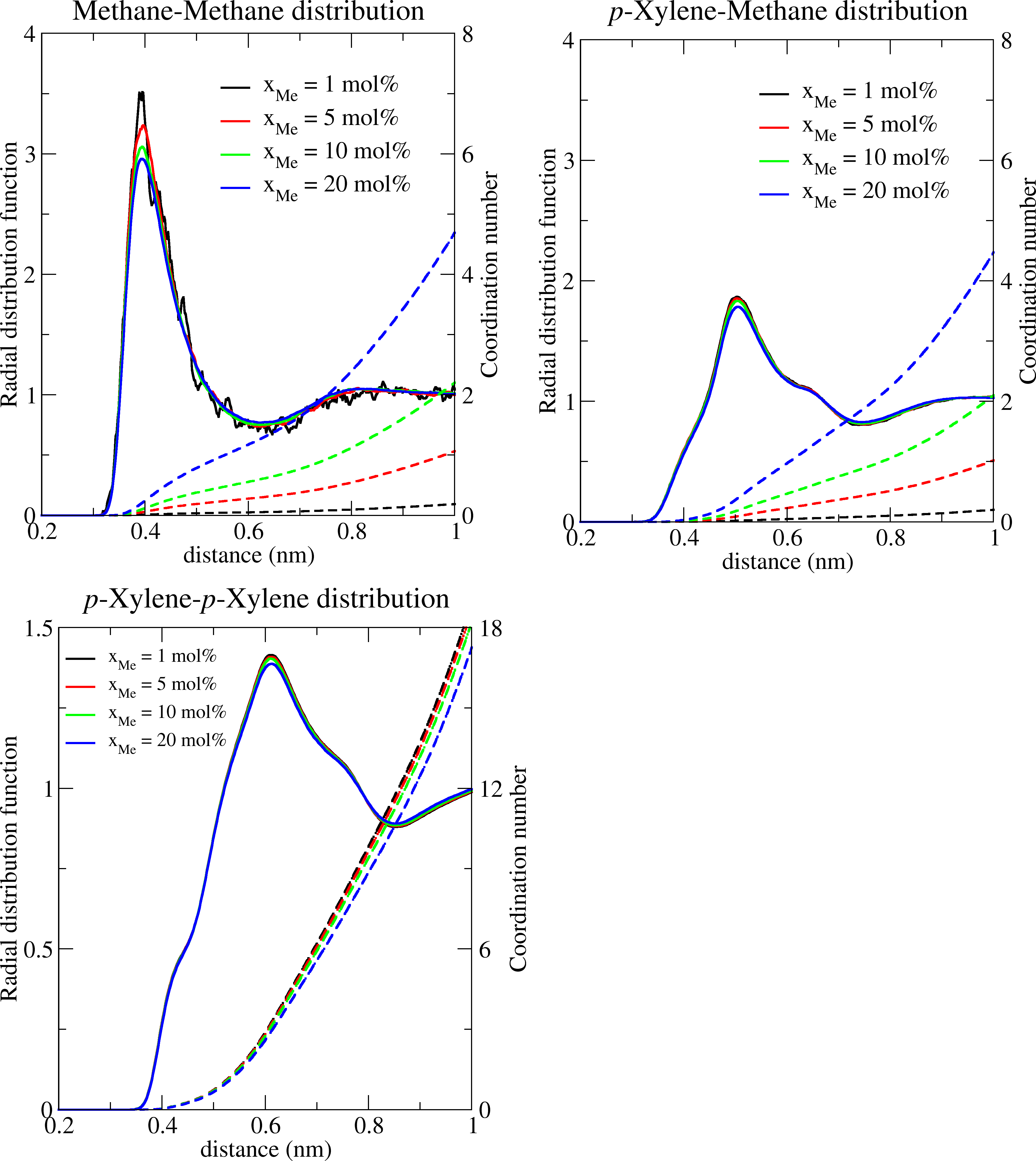
Figure S4. Evolution of the radial distribution function (RDF, full lines, left axis) and running coordination numbers (dashed lines, right axis) for methane-methane, methane-*p*-xylene, and *p*‑xylene- *p*-xylene with increasing methane molar fraction (from 1 to 20 mol.%) in *p*‑xylene liquid. These solution structures represent the homogeneous bulk phase of systems at 298 K and an external pressure of 45 bar.

**REFERENCES**

1 DIPPR Project 801 - Full Version, https://app.knovel.com/hotlink/toc/id:kpDIPPRPF7/dippr-project- 801-full/dippr-project-801-full, accessed 16. 8. 2024
